# Cardiovascular System Modeling

**DOI:** 10.1155/2012/583172

**Published:** 2012-12-30

**Authors:** Ling Xia, Alan Murray, Dingchang Zheng, Feng Liu, Xuesong Ye, Gangmin Ning

**Affiliations:** ^1^Department of Biomedical Engineering, Zhejiang University, Hangzhou 310027, China; ^2^Cardiovascular Physics and Engineering Research Group, Institute of Cellular Medicine, Newcastle University, Freeman Hospital, Newcastle upon Tyne NE7 7DN, UK; ^3^School of Information Technology and Electrical Engineering, The University of Queensland, Brisbane, QLD 4072, Australia

Mathematical models and numerical simulations of the cardiovascular system are very useful for understanding the mechanisms influencing its function and its physiological and pathological processes. This modeling research increases the potential for developing new diagnostic and therapeutic cardiovascular techniques or devices and is also helpful for pharmacological research [1]. The recent development of computational methods to solve modeling difficulties in diverse disciplines such as systems biology and computer science led to the idea of compiling a special volume on the subject.

This special issue mainly focuses on computational and mathematical methods in cardiovascular system modeling. The research papers cover the topics of cellular or subcellular cardiac cell modeling, cardiovascular anatomical structure modeling, cardiac electrophysiology modeling and simulation, cardiac mechanics modeling and analysis, heart failure modeling and simulation, GPU-based cardiac computational technology, modeling and simulation of the vascular system and mechanics, and computational methods in cardiovascular imaging. In the following, we briefly summarize the twelve papers included in this issue. 

Calcium dynamics is very important in cardiac cell modeling and is often modeled using deterministic ordinary differential equations (ODEs). However, is it appropriate to model the dynamics of the subspace calcium using deterministic ODEs? Do we require a stochastic description that accounts for the fundamentally discrete nature of the calcium-regulated calcium influx? To answer these questions, S. H. Weinberg and G. D. Smith constructed and analyzed a minimal Markov model of a calcium-regulated calcium channel and associated subspace and also compared the expected steady-state subspace calcium concentration in the stochastic model (a result that accounts for the small subspace volume) with the corresponding deterministic ODE model (an approximation that assumes large system size).

L. Lu et al. developed a coupled calcium dynamics model by integrating the spatiotemporal Ca^2+^ reaction-diffusion system into the cellular electrophysiological model. Their model was applied to study the effect of rogue RyRs on Ca^2+^ cycling and membrane potential in failing heart. Their simulation showed that rogue RyR with tiny Ca^2+^ release flux is an important factor in triggering arrhythmia in failing cardiac cells and suggested that rogue RyRs could influence the initiation of Ca^2+^ release events (especially Ca^2+^ waves) and consequently delayed afterdepolarizations or triggered action potentials.

Modeling and simulation of the high complexity of the cardiac electrophysiological processes and the detailed microstructure of cardiac tissue face the challenges of the high computational cost. B. G. de Barros et al. developed a cardiac electrophysiological model using a very fine spatial discretization (8 *μ*m) and a complex cell model based on Markov chains for the characterization of ion channel's structure and dynamics. Multi-GPU platform was then used to compute parallelly. The execution time of the simulations was reduced from over 6 days (on a single processor) to 21 minutes (on a small 8-node cluster equipped with 16 GPUs, i.e., 2 GPUs per node). 

D. Deng et al. developed a human heart model with detailed anatomical structure, conduction system, and experimentally measured fiber orientations. Such detailed anatomical heart model could be very useful for a better understanding of the mechanisms influencing cardiovascular function and its physiological and pathological processes.

Electromechanical modeling of the heart is one of the hottest topics in cardiovascular system modeling. H. Xia et al. presented a fully coupled electromechanical model of a dog heart. This model integrated cardiac electrophysiology and cardiac mechanics through excitation-induced contraction and deformation-induced current. It provides a useful tool to understand cardiovascular dynamics. J. Dou et al. presented a mechanical optimization strategy for cardiac resynchronization therapy (CRT) based on an electromechanical heart model. This mechanical-based optimization approach, in combination with the electrical-based approach, provided a more reasonable method for optimization of lead positions and pacing delays of CRT.

M. Jiang et al. reported a new approach for solving the ECG inverse problem. The inverse problem was treated as a regression problem with multiinputs (body surface potentials (BSPs)) and multioutputs (transmembrane potentials (TMPs)) and was then solved by a maximum margin clustering-support vector regression method. Their new approach achieved a good performance in reconstructing TMPs from BSPs and could be useful for noninvasive electrocardiographic imaging [2]. 

In the area of cardiac mechanics and the vascular system, in order to simulate the Frank-Starling law of the heart, S. Ribarič and M. Kordaš developed a lumped parameter model with a vascular circuit based on a frog heart with one atrium and one ventricle. This model could be used to study the basic concept of cardiovascular physiology in a macroscopic manner, for example, the time course of atrial and ventricular pressure during systole and diastole, the rate of myocardial contraction and relaxation, and so on. A similar model was used by M. Sever et al. to simulate the exercise-induced syncope in patients with severe aortic valve stenosis, and the effects of controlled change in heart rate and the ventricular contractility or systemic vascular resistance on the cardiac hemodynamics were investigated.

F. Z. Boroujeni et al. presented an improved center-line tracing algorithm for automatic extraction of the coronary arterial tree based on the second-order local features. This algorithm avoided the limitations in handling highly curved segments and sudden changes of vessel diameter at the site of arterial lesions and thus could be more suitable for feature extraction and quantitative coronary analysis in real applications with inherently noisy data.

J. Ding et al. investigated the hemodynamic effect of competitive flow caused by different degrees of left anterior descending (LAD) artery stenosis in internal thoracic artery (ITA) bypass graft. An idealized ITA-LAD model was developed, and the simulation suggested that the coronary bypass graft surgery should preferentially be carried out when the LAD stenosis is higher than 75%. This study is useful for guiding the treatment of proximal LAD stenosis.

Y. Ren et al. presented a fast parameters estimation algorithm to construct a cardiovascular model for the evaluation of drugs used for the treatment of heart failure (HF). The model is able to predict hemodynamic conditions of HF patients undergoing treatment. Also, a novel comprehensive index was produced to assess the outcome of HF treatment. This study offers a quantitative tool for having a patient-specific HF treatment plan and is useful in evaluating the dose effect of HF drugs.

In summary, the twelve research papers in the special issue summarize the most recent developments and ideas in the field of cardiovascular system modeling. They are worth reading by the researchers who are working in the cardiovascular modelling or related fields. 

*Ling Xia* *Alan Murray* *Dingchang Zheng* *Feng Liu* *Xuesong Ye* *Gangmin Ning*
